# Supplementary material for: Smooth Interpolating Curves with Local Control and Monotone Alternating Curvature
Source: Comput Graph Forum. 2022 Oct 6;41(5):25–38. doi: 10.1111/cgf.14600 (PMC9827861; doi:10.1111/cgf.14600)
Supplement: Supplementary file 1 — Supplement Material [file CGF-41-25-s001.zip › Local-Smooth-Interpolating-MonoCurvature/extern/clothoids/submodules/Utils/docs/api-cpp/program_listing_file_Utils_Trace.hxx.html]

Program Listing for File Trace.hxx — Utils v1.2

### Navigation

- index
- toc
- Utils »
- Program Listing for File Trace.hxx

# Program Listing for File Trace.hxx¶

↰ Return to documentation for file (`Utils/Trace.hxx`)

```
/*--------------------------------------------------------------------------*\
 |                                                                          |
 |  Copyright (C) 2017                                                      |
 |                                                                          |
 |         , __                 , __                                        |
 |        /|/  \               /|/  \                                       |
 |         | __/ _   ,_         | __/ _   ,_                                |
 |         |   \|/  /  |  |   | |   \|/  /  |  |   |                        |
 |         |(__/|__/   |_/ \_/|/|(__/|__/   |_/ \_/|/                       |
 |                           /|                   /|                        |
 |                           \|                   \|                        |
 |                                                                          |
 |      Enrico Bertolazzi                                                   |
 |      Dipartimento di Ingegneria Industriale                              |
 |      Universita` degli Studi di Trento                                   |
 |      email: enrico.bertolazzi@unitn.it                                   |
 |                                                                          |
\*--------------------------------------------------------------------------*/


#pragma once

#ifndef DOXYGEN_SHOULD_SKIP_THIS

#ifndef UTILS_TRACE_dot_HH
#define UTILS_TRACE_dot_HH

#ifndef UTILS_ERROR0
  #define UTILS_ERROR0(MSG) \
  throw Utils::Runtime_Error( MSG, __FILE__, __LINE__ )
#endif

#ifndef UTILS_ASSERT0
  #define UTILS_ASSERT0(COND,MSG) if ( !(COND) ) UTILS_ERROR0( MSG )
#endif

#ifndef UTILS_WARNING0
  #define UTILS_WARNING0(COND,MSG) if ( !(COND) ) std::cerr << MSG
#endif

#ifndef UTILS_ERROR
  #define UTILS_ERROR(...) \
  throw Utils::Runtime_Error( fmt::format(__VA_ARGS__), __FILE__, __LINE__ )
#endif

#ifndef UTILS_ASSERT
  #define UTILS_ASSERT(COND,...) if ( !(COND) ) UTILS_ERROR( __VA_ARGS__ )
#endif

#ifndef UTILS_WARNING
  #define UTILS_WARNING(COND,...) if ( !(COND) ) fmt::print( __VA_ARGS__ )
#endif

#ifndef UTILS_ERROR_TRACE0
  #define UTILS_ERROR_TRACE0(MSG) \
  throw Utils::Runtime_TraceError( MSG, __FILE__, __LINE__ )
#endif

#ifndef UTILS_ASSERT_TRACE0
  #define UTILS_ASSERT_TRACE0(COND,MSG) if ( !(COND) ) UTILS_ERROR_TRACE0( MSG )
#endif

#ifndef UTILS_ERROR_TRACE
  #define UTILS_ERROR_TRACE(...) \
  throw Utils::Runtime_TraceError( fmt::format(__VA_ARGS__), __FILE__, __LINE__ )
#endif

#ifndef UTILS_ASSERT_TRACE
  #define UTILS_ASSERT_TRACE(COND,...) if ( !(COND) ) UTILS_ERROR_TRACE( __VA_ARGS__ )
#endif

#ifdef UTILS_NO_DEBUG
  #ifndef UTILS_ASSERT0_DEBUG
    #define UTILS_ASSERT0_DEBUG(COND,MSG)
  #endif
  #ifndef UTILS_ASSERT_DEBUG
    #define UTILS_ASSERT_DEBUG(COND,...)
  #endif
#else
  #ifndef UTILS_ASSERT0_DEBUG
    #define UTILS_ASSERT0_DEBUG(COND,MSG) UTILS_ASSERT0(COND,MSG)
  #endif
  #ifndef UTILS_ASSERT_DEBUG
    #define UTILS_ASSERT_DEBUG(COND,...) UTILS_ASSERT(COND,__VA_ARGS__)
  #endif
#endif

#endif

#ifdef __GNUC__
#pragma GCC diagnostic push
#pragma GCC diagnostic ignored "-Wpadded"
#endif
#ifdef __clang__
#pragma clang diagnostic push
#pragma clang diagnostic ignored "-Wpadded"
#endif

namespace Utils {

  #ifndef DOXYGEN_SHOULD_SKIP_THIS
  using std::basic_ostream;
  using std::string;
  using std::runtime_error;
  #endif

  typedef basic_ostream<char> ostream_type;

  void
  printTrace(
    int                line,
    char const * const file,
    string const     & msg,
    ostream_type     & stream
  );

  class Runtime_TraceError : public runtime_error {
  private:
    string
    grab_backtrace(
      string const &     reason,
      char const * const file,
      int                line
    ) const;

  public:
    explicit
    Runtime_TraceError( string const & reason, char const * const file, int line )
    : runtime_error( grab_backtrace( reason, file, line ) )
    { }

    virtual const char* what() const noexcept override;
  };

  class Runtime_Error : public runtime_error {
  public:
    explicit
    Runtime_Error( string const & reason, char const * const file, int line )
    : runtime_error( fmt::format( "\n{}\nOn File:{}:{}\n", reason, file, line ) )
    { }

    explicit
    Runtime_Error( char const * const reason, char const * const file, int line )
    : runtime_error( fmt::format( "\n{}\nOn File:{}:{}\n", reason, file, line ) )
    { }

    virtual const char* what() const noexcept override;
  };

}

#endif
```

### Quick search

### Table of Contents

- rang - Colors for your Terminal
- Zstr
- C++ API

«
hide menu

menu
sidebar
»

### Navigation

- index
- toc
- Utils »
- Program Listing for File Trace.hxx

© Copyright 2021, Enrico Bertolazzi.
Created using Sphinx 4.2.0.
